# Supplementary material for: In situ atomic-scale observation of dislocation climb and grain boundary evolution in nanostructured metal
Source: Nat Commun. 2022 Jul 18;13:4151. doi: 10.1038/s41467-022-31800-8 (PMC9293973; doi:10.1038/s41467-022-31800-8)
Supplement: Supplementary file 1 — Supplementary Information [file 41467_2022_31800_MOESM1_ESM.pdf]

Supplementary Materials for

In situ atomic-scale observation of dislocation climb and grain boundary evolution in nanostructured metal

Shufen Chu[1], Pan Liu[1,2]*, Yin Zhang[3], Xiaodong Wang[1], Shuangxi Song[1], Ting Zhu[3]*, Ze Zhang[4], Xiaodong Han[5], Baode Sun[1], Mingwei Chen[2,6]*

*Corresponding authors: panliu@sjtu.edu.cn (PL); ting.zhu@me.gatech.edu (TZ); mwchen@jhu.edu (MC)

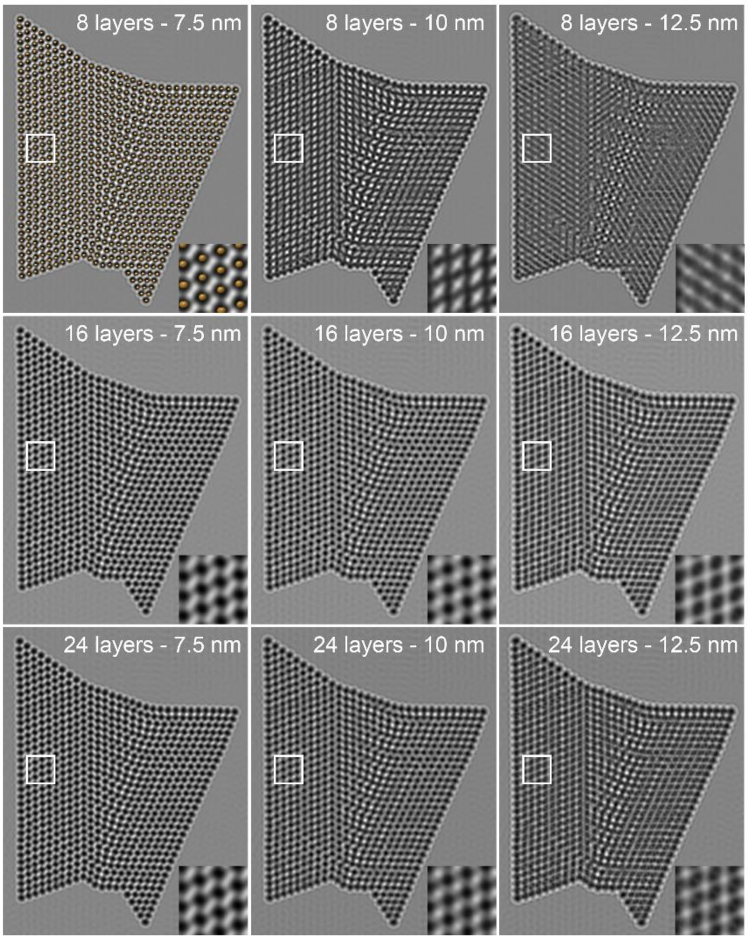

Supplementary Figure 1. A series of simulated HRTEM images of the Au ligament. Sample thicknesses and defocus values are given in the corresponding images. A structure model is overlapped with the top left image, indicating that with appropriate parameters, the dark spots represent Au atomic columns while the white spots represent channels between atomic columns. The insets show the magnified images of the white squared regions, demonstrating details of the simulated results. The simulated images vary dramatically for different defocus values in an increment of 2.5 nm as well as different sample thicknesses. By refining all the parameters, the sample thickness of 16 layers of $(1\bar{1}0)$ atomic plane (i.e., 4.8 nm) and the defocus value of 10 nm are determined to make the simulated image provide a best fit to the experimental images.

Supplementary Note 1. The structure model for image simulations was reconstructed based on the HRTEM image of the Au ligament at $t = 0$ s in Fig. 1a. The possible sample thicknesses were set as 8, 16, and 24 layers of $(1\bar{1}0)$ atomic plane, which corresponded to approximately 2.4 nm, 4.8 nm, and 7.1 nm, respectively. HRTEM image simulations were conducted using the commercial xHREM software (HREM RESEARCH INC.), which emerges from the image simulation programs based on the FFT multislice technique developed by Ishizuka[1,2]. The detailed parameters used in the image simulations are given in **Supplementary Table 1**.

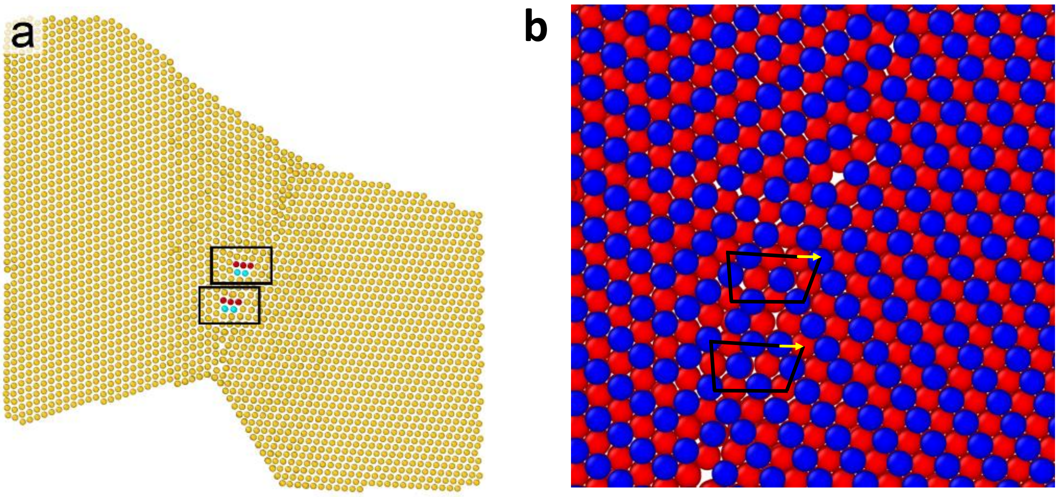

Supplementary Figure 2. Full Burgers vectors of the dislocations determined through an atomistic model. (a) Atomistic model of the Au ligament reconstructed from the HRTEM image in Fig. 1a. Atoms in the two neighboring dislocation cores are colored and boxed. (b) Magnified image of the two dislocation cores boxed in (a). The red and blue atoms represent two alternating $(\bar{1}10)$ layers perpendicular to the zone axis. From the Burgers circuit analysis (non-closure black lines), the Burgers vectors of the two dislocations are marked by the yellow arrows, respectively. Note that the yellow Burgers vector of the top dislocation points from the red to blue atom, while that of the bottom dislocation from the blue to red. This indicates that the out-of-plane screw components of the two Burgers vectors have the opposite signs and they are determined as $1/4\,[1\bar{1}0]$ and $1/4\,[\bar{1}10]$, respectively. Thus, the full Burgers vectors of these dislocations can be identified as $1/2[011](11\bar{1})$ or $1/2[101](11\bar{1})$.

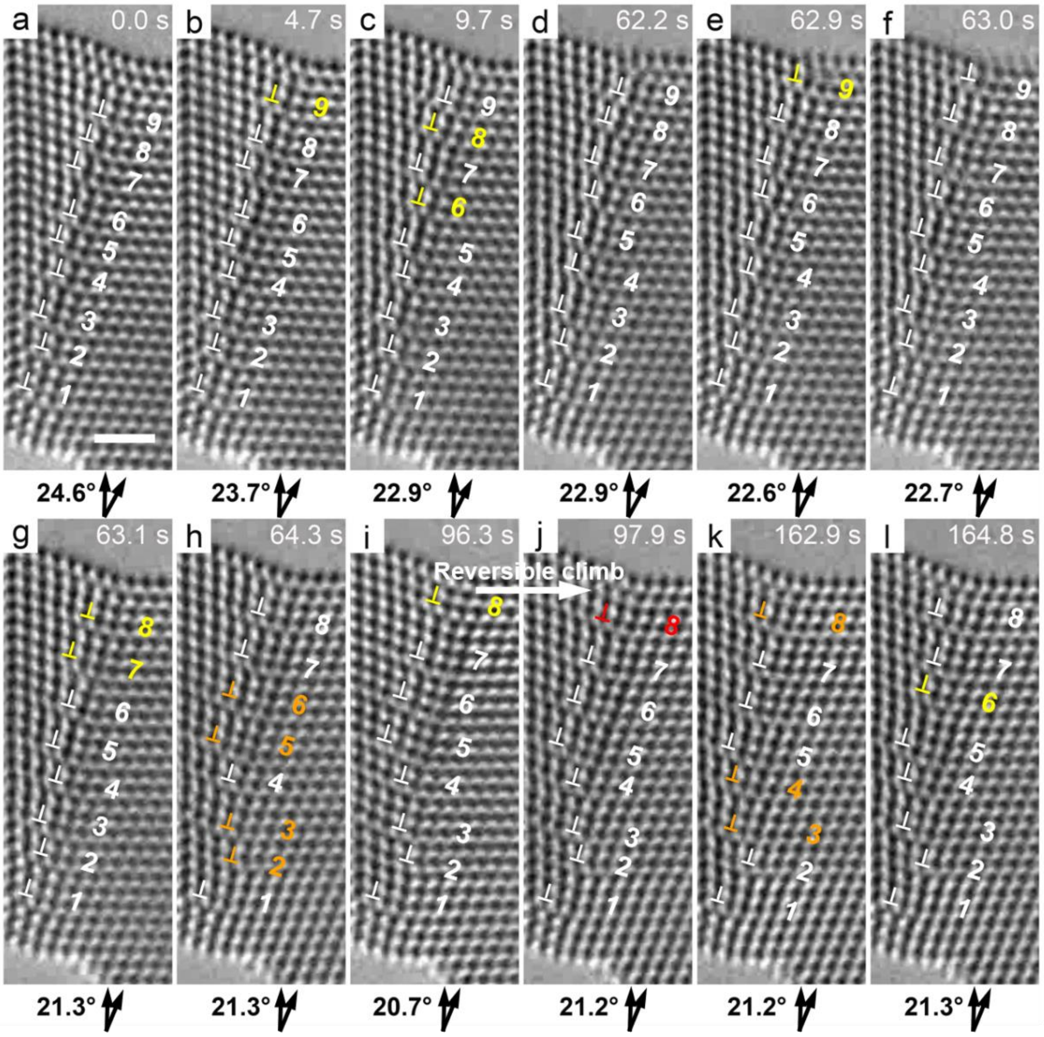

Supplementary Figure 3. *In situ* HRTEM images showing detailed GB dislocation behaviors. (a)-(l) Each event of positive and negative climb in the present frame relative to the previous frame is marked by a yellow and a red symbol ⊥, respectively, while dislocations staying still are marked by white symbols ⊥. Each event of dislocation glide is marked by an orange symbol ⊥. Reversible climb occurred in (i) and (j), likely due to local stress fluctuation. Scale bar: 1 nm.

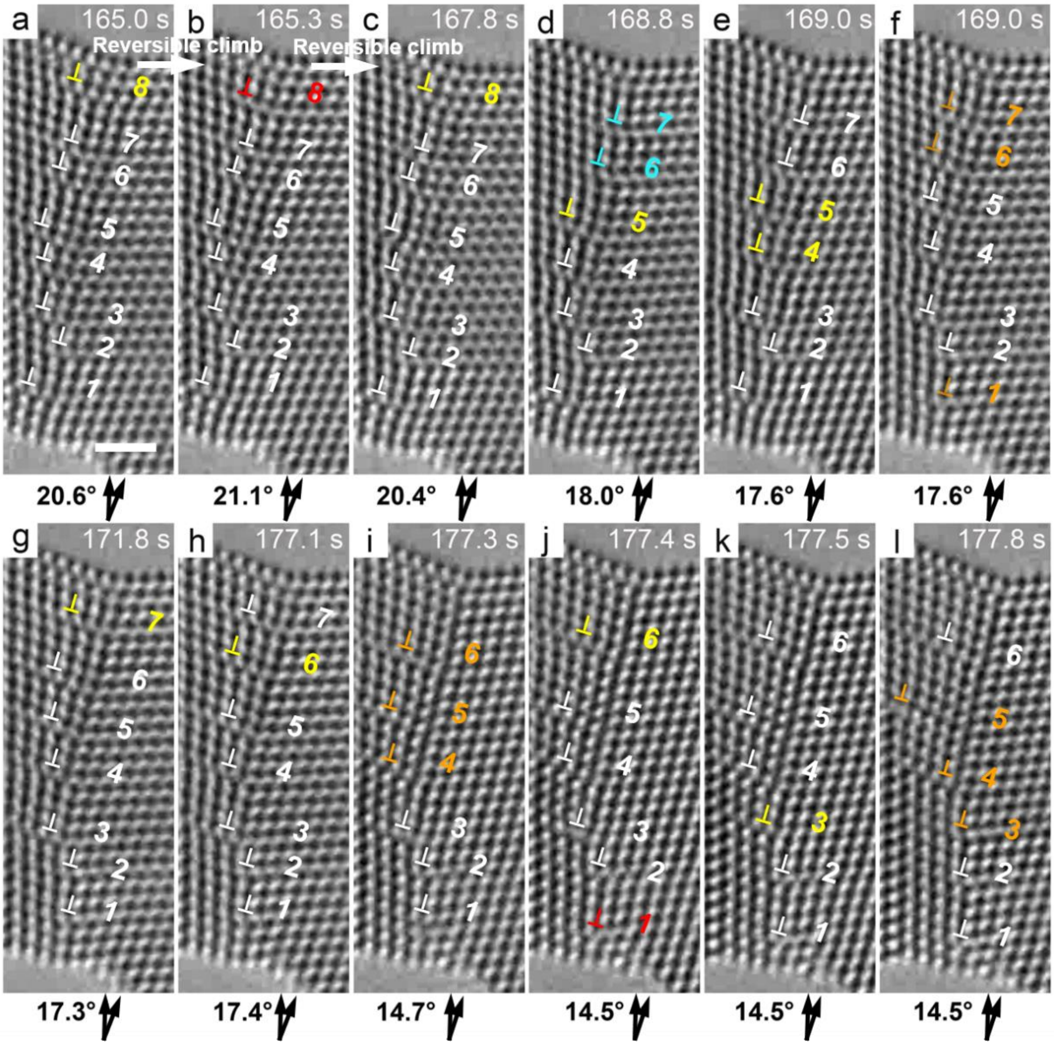

Supplementary Figure 4. ***In situ*** **HRTEM images showing detailed GB dislocation behaviors.** (a)-(l) The convention of symbols is the same as Supplementary Fig. 3. Reversible climb of GB dislocations occurred in (a)-(c). Coupled dislocation climb and sliding are marked by cyan symbols ⊥. Scale bar: 1 nm.

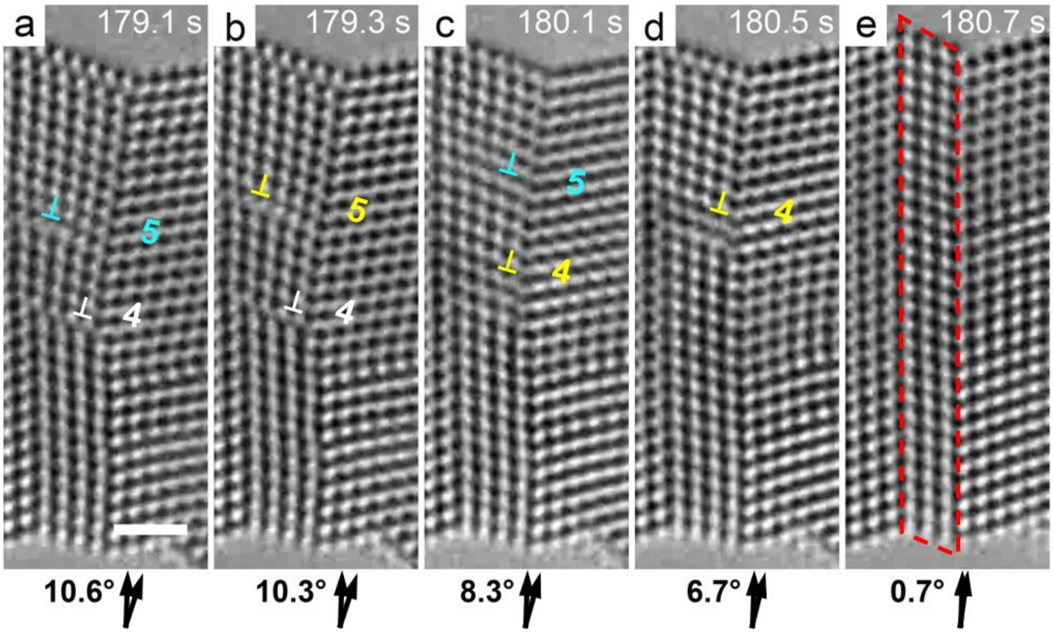

Supplementary Figure 5. ***In situ*** **HRTEM images showing detailed GB dislocation behaviors.** (a)-(e) Climb and annihilation of dislocation "4" and "5". The HAGB eventually evolved into a Σ3 coherent TB in (e). The thickness of the twin lamella decreased from 5 layers of {111} atomic plane in (d) to 4 layers in (e), and this could be ascribed to TB migration under local shear stress vibrations. Scale bar: 1 nm.

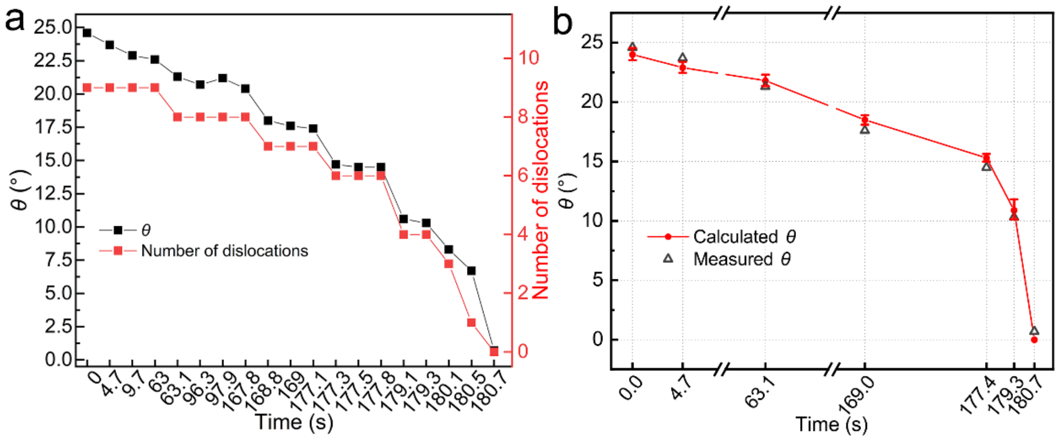

Supplementary Figure 6. Quantitative analysis of misorientation angle and number of dislocations. (a) Misorientation angle θ and the number of GB dislocations are plotted against time. (b) Calculated θ by the Frank's equation[3] and the measured θ as a function of time, showing close agreement. The error bars are given on the calculated θ considering the measurement errors of the dislocation spacings.

Supplementary Note 2. For an arbitrary large GB with an angle of θ, the relationship between θ and the total Burgers vector **B** of the GB dislocations can be written as the Frank's equation[3]

$$\mathbf{B} = 2\sin\frac{\theta}{2}(\mathbf{P} \times \mathbf{a}) \tag{1}$$

Where **P** is a probe vector in the GB orthogonal to **B** (here **P** corresponds to the vector **V** in Equation (19-14) in *Theory of dislocations* (2nd Edition, by Hirth and Lothe)). **a** is a unit vector along the axis of rotation. According to Equation (1), a decrease in the number of the GB dislocations would lead to decreased **B**, and consequently decreased GB angle θ. In our experimental observations, GB dislocation climb is responsible for in-plane grain rotation. Thus, **P** is perpendicular to **a**. The equation can be written as[3,4]:

$$\frac{|\mathbf{B}|}{|\mathbf{P}|} = 2\sin\frac{\theta}{2} \tag{2}$$

$|\mathbf{B}|$ can be calculated by multiplying the edge components of the GB dislocations (0.25 nm) with the number of the GB dislocations. $|\mathbf{P}|$ can be obtained by measuring the total dislocation spacing. The topmost dislocation is not considered for its unknown dislocation spacing. We can then compare the calculated θ by Equation (2) with our experimental measurements. For example, at $t = 0$ s, $|\mathbf{B}|$ is calculated to be 2.0 nm and the total dislocation spacing $|\mathbf{P}|$ is 4.8 nm. The calculated θ is 24.0°, which is consistent with the measured angle θ (24.6°). As shown in Supplementary Fig. 6, the calculated θ always closely agrees with the measured θ during *in situ* straining. It should be noted that only the edge components of the Burgers vectors which are perpendicular to the tilt GB plane are considered because the screw components which are parallel to the GB plane are undetectable in the HRTEM images and they do not contribute to the tilt angle of the GB.

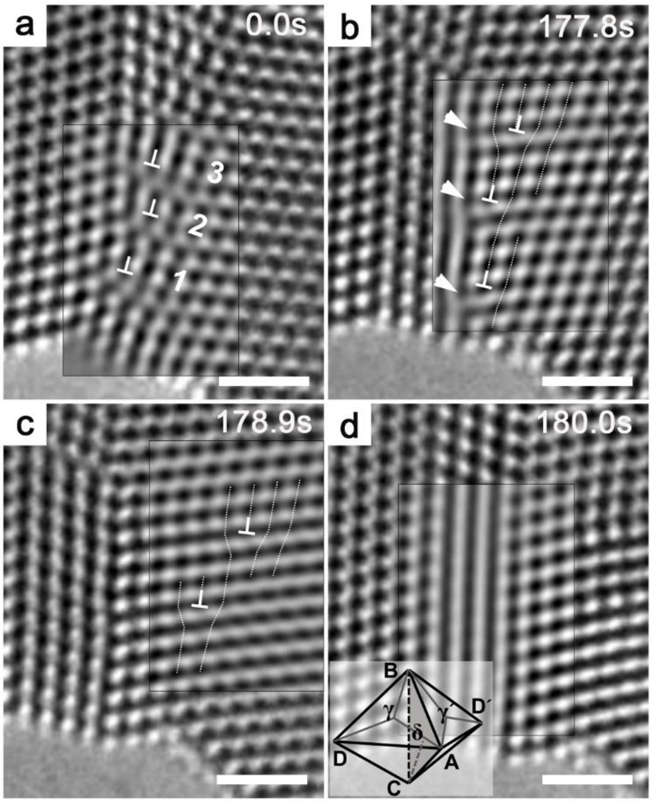

Supplementary Figure 7. Interaction between dislocations “1” - “3” and the TB. Dislocations “1” - “3” in (a) passed through the TB to enter the right grain, resulting in migration of the TB to the right. Three steps were produced in (b) as marked by white arrows; they were the projection of 1/6[$\bar{1}2\bar{1}$] partial dislocations. The reaction between each dislocation and the TB followed 1/2[011] → 1/2[$\bar{1}0\bar{1}$] + 1/6[$\bar{1}2\bar{1}$] (DA→A´D´+ Cδ)[5,6]. As a result of this reaction, each full dislocations (A´D´) glided into the right grain, while each twin partial (Cδ) glided along the TB and vanished in (c), resulting in migration of the TB to the right. A perfect twin lamella formed in (d). To display the defects more clearly, the boxed regions were filtered by the inverse Fast Fourier Transform method. Scale bar: 1 nm.

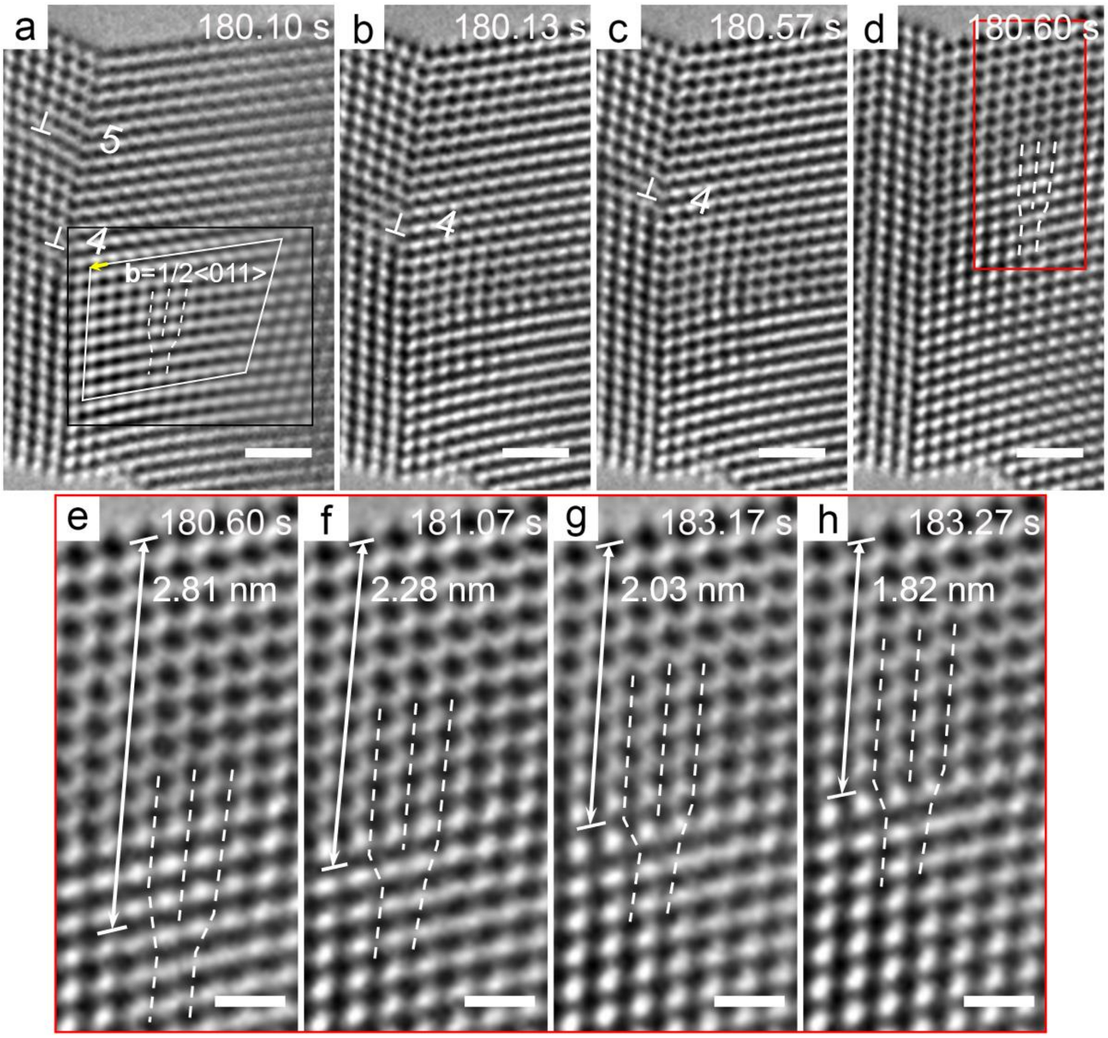

Supplementary Figure 8. A short time series of HRTEM images right before and after dislocations "5" and "4" disappear. (a)-(d) Because of fast dislocation activities involved, the detailed processes of their disappearance were not captured. However, it can be deduced from the movements of surrounding dislocations that dislocations "5" and "4" annihilated from the top surface of the ligament by climb, since no new dislocation was found at the TB or in the right grain. In (a), a Burgers circuit encloses a full dislocation in the right grain. To display the dislocation more clearly, the black boxed regions were filtered by the inverse Fast Fourier Transform method. This dislocation resulted from slip transmission of a dislocation (in the middle grain) across the TB and it further climbed upward in the right grain as indicated by the red box in (d). (e)-(h) Zoom-in images of the red-boxed region in (d) showing dislocation climb in the lattice of the right grain. The distance between the dislocation core and the top surface is indicated by a double-headed arrow, and this distance decreased from 2.81 nm (e) to 1.82 nm (h), evidencing the lattice dislocation climb. Scale bar: 1 nm in (a)-(d), and 0.5 nm in (e)-(h).

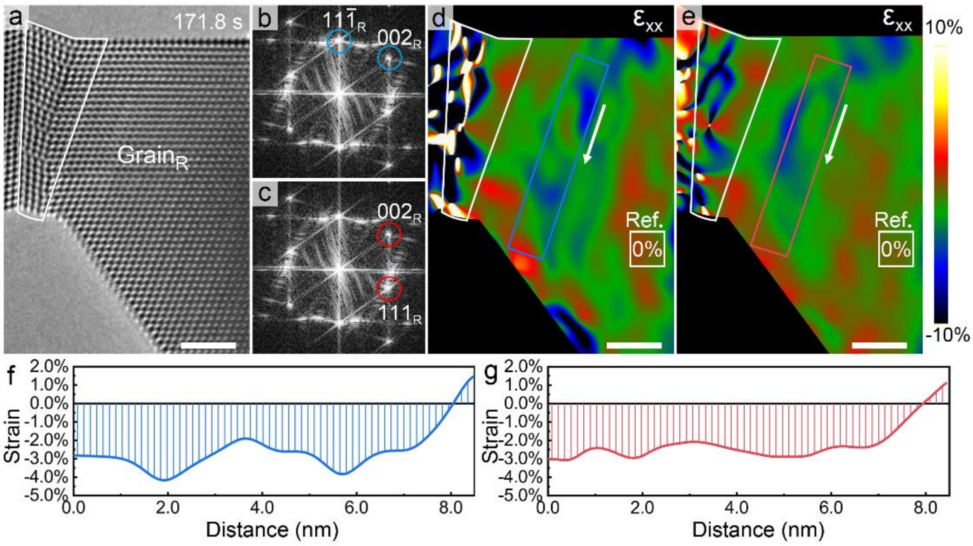

Supplementary Figure 9. Lattice strain maps calculated by GPA method with different choices of g-vectors. (a) HRTEM image of the Au ligament at 171.8 s. The HAGB region is marked by a white polygon. (b)-(c) FFT images of the ligament. Different sets of **g**-vectors are circled in blue and red, respectively. (d)-(e) Lattice strain maps in (d) and (e) corresponding to the selected **g**-vectors in (b) and (c), respectively. The strain color map spans a range from -10% (black) to 10% (white). (f)-(g) Profiles of strain values on the GB plane extracted from the rectangles in (d) and (e) along the arrows, respectively. The strain maps and the corresponding profiles indicate the compressive and tensile lattice strain on the upper and lower part of the GB plane, respectively. Scale bar: 2 nm.

Supplementary Note 3. The GPA method was applied to the lattice of the right grain rather than to the interface. In other words, the strain distribution of the lattice near the GB was analyzed to investigate the mechanical loading on the ligament. The GPA results are sensitive to the choice of parameters in the mapping. We discuss the influence of the choice of **g**-vectors and reference frame on the GPA results in detail. In Supplementary Fig. 9, as denoted by the white squares in (d) and (e), the same regions are selected as reference frames to compare the influence of the choice of **g**-vectors. The reference frames are far from the GB region and are located in the lower middle of the ligament (i.e., almost on the neutral plane) so that the strain in the reference regions is nearly zero. It is clear that strain maps with different choices of **g**-vectors show similar strain distributions of the right grain. That is to say, the choice of **g**-vectors does not affect the GPA results. However, it is necessary to select the non-parallel **g**-vectors belonging to the right grain. Supplementary Fig. 10 show lattice strain maps of ε_{yy} and ε_{xy} using the **g**-vectors selected in Supplementary Fig. 9b.

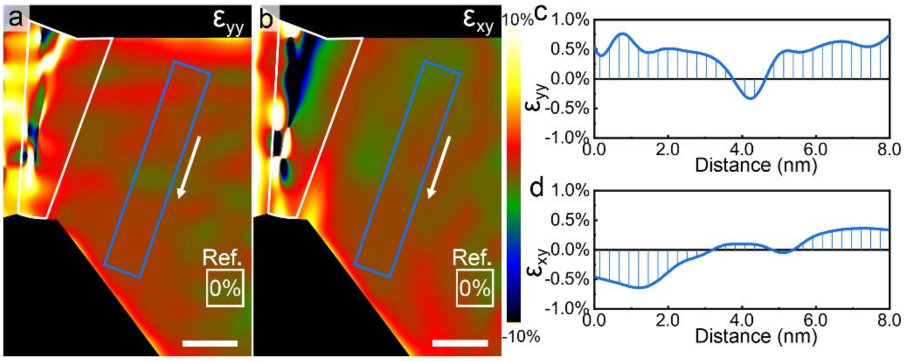

Supplementary Figure 10. Lattice strain maps of ε_{yy} and ε_{xy} (a and b) and the corresponding profiles of strain value extracted from the rectangles (c and d). In general, both strain distributions are uniform in the right grain. Both values of ε_{yy} and ε_{xy} are close to zero, ranging from ~-0.5% to ~0.5%. Scale bar: 2 nm.

To discuss the effect of the choice of reference frame on the GPA results, we set the reference frame in the region that deviates from the original position and compared the changes in the strain distribution. Supplementary Fig. 11a shows the strain map in Supplementary Fig. 9d. Although it may not be a "true" zero-strain region, the reference frame is placed far from the GB region in the lower middle of the ligament, i.e., on the neutral plane, so that the strain within the region is very close to zero. The profile of the strain value extracted from the rectangle is shown below the strain map. The crossover from compressive to tensile strain is marked with a red line. In Supplementary Fig. 11b, the reference frame (marked by the solid line box) is located above the previous position (marked by the dashed box) but still as far away from the GB region as possible. It can be seen from the profile that as the reference frame is positioned away from the neutral plane and may be affected by residual compressive stress, the strain value is generally increased and the crossover from compressive to tensile strain slightly deviates from its original position. Conversely, the reference frame is placed below the previous position in Supplementary Fig. 11c. As shown in the profile, probably affected by residual tensile stress, the strain value decreases and the crossover also deviates from the original position. In summary, the choice of reference frame has a large effect on the absolute value of strain, but does not affect the relative value of strain on the GB plane. Importantly, the lattice strain always changes from compression to tension on the GB plane from the top to bottom, which is consistent with the distribution of the applied bending stress.

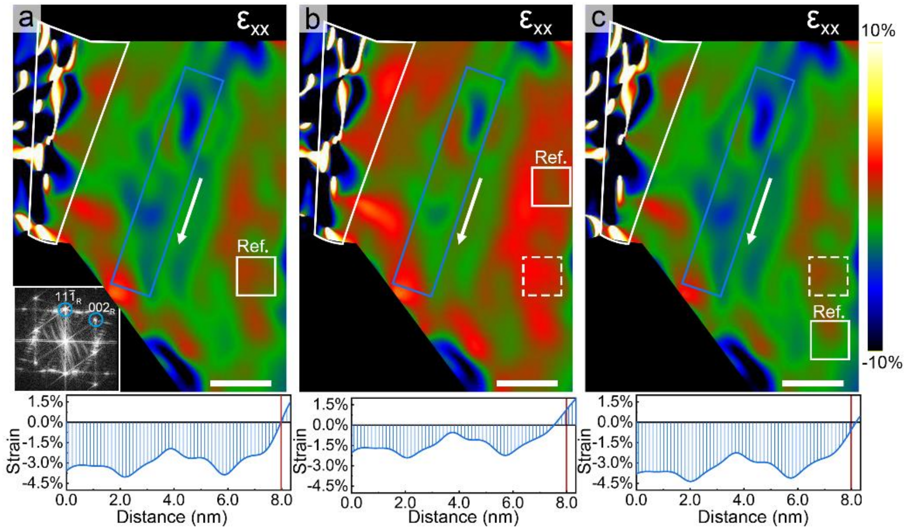

Supplementary Figure 11. Lattice strain maps with different choices of reference frame and the corresponding profiles of strain value. (a)-(c) The reference frames are set at the white boxed regions with solid lines. The crossovers from compressive to tensile strain in the strain profiles are marked with a red line. The selected **g**-vectors are circled in blue in the FFT image in the inset of (a). Scale bar: 2 nm.

In the GPA analysis, a Gaussian smoothing parameter of 5.0 was applied. Masks with radii of 1/5 of the corresponding **g**-vectors were used for generating the lattice strain maps. The mask size shows the area selected in the Fourier space around the spot of interest and the inverse value of the selected spot corresponds to the effective spatial resolution of the lattice strain maps. It is worth noting that the spatial resolution and the precision are roughly inversely proportional to each other, and a compromise between them must be made to obtain reliable lattice strain maps.

Supplementary Note 4. In the interpretation of HRTEM images, the small sample thickness and minimized spherical aberration make it possible to directly link the images with the projection of crystal structures based on the a charge density project approximation[7]. For the phase-contrast HRTEM, the image contrast formation can be described by the phase contrast transfer function (CTF) U(u), which can be written as

$$\mathrm{U}(u) = \exp(\mathrm{i}(u)) = \exp(\mathrm{i}\pi\Delta f\lambda u^2 + 0.5\mathrm{i}\pi \mathrm{C_s}\lambda^3 u^4) \quad (3)$$

where Δf and Cs correspond to a defocus value and a spherical aberration coefficient of the objective lens, λ is the electron wave length and u is the spatial frequency. For the Cs-corrected TEM, the Cs value is small and can be ignored (i.e., Cs ~ 0). The CTF can be re-written as:

$$\mathrm{U}(u) = \exp(\mathrm{i}(u)) = \exp(\mathrm{i}\pi\Delta f\lambda u^2) \quad (4)$$

When the defocus value Δf is small,

$$\mathrm{U}(u) = 1 + \mathrm{i}\pi\Delta f\lambda u^2 \quad (5)$$

For a thin TEM specimen, the specimen transfer function f(x, y) can be simplified by the weak phase-object approximation:

$$\mathrm{f}(x, y) = \exp(\mathrm{i}\sigma \mathrm{v}(x, y)) \quad (6)$$

where the $v(x, y)$ is the electrostatic potential and σ is the interaction constant. Then, the CTF modulated wave function on the back-focus plane of the objective lens is given by:

$$F(x, y) = \exp(i\pi\Delta f\lambda u^2) \cdot FT\{f(x, y)\} = \exp(i\pi\Delta f\lambda u^2) \cdot F(u) \quad (7)$$

By inverse Fourier Transform (FT), the wave function at the image plane of the objective lens becomes:

$$\Psi(x, y) = f(x, y) + i\pi\Delta f\lambda FT\{u^2 \cdot F(u)\} \quad (8)$$

Based on FT differential property, the wave function can be written as:

$$\Psi(x, y) = \{1 - \frac{\Delta f\lambda\sigma\nabla^2 v(x, y)}{4\pi} + \frac{i\Delta f\lambda\sigma^2}{4\pi}[(\frac{\partial v(x, y)}{\partial x})^2 + (\frac{\partial v(x, y)}{\partial y})^2]\}\exp(-i\sigma v(x, y)) \quad (9)$$

The intensity distribution on the image plane can be derived from the wave function:

$$I(x, y) = \Psi(x, y)\Psi^*(x, y) = 1 - \frac{\Delta f\lambda\sigma\nabla^2 v(x, y)}{2\pi} \quad (10)$$

The relation between electrostatic potential $v(x, y)$ and projected charge density $\rho(x, y)$ can be described by Poisson equation:

$$\nabla^2 v(x, y) = -4\pi\rho(x, y) \quad (11)$$

Then,

$$I(x, y) = 1 + 2\Delta f\lambda\sigma\rho(x, y) \quad (12)$$

Consequently, the image contrast $c(x, y)$ is:

$$c(x, y) = 2\Delta f\lambda\sigma\rho(x, y) \quad (13)$$

which is linearly proportional to the charge densities of atoms and molecules on the projection plane. Since the projected charge density function $\rho(x, y)$ is positively correlated with the sample thickness, a positive correlation between the image contrast and the sample thickness (i.e., the number of atoms in an atomic column) can be established when the TEM sample is sufficiently thin. This provides a theoretical basis for our semi-quantitative analysis of HRTEM images relating local contrast change with atom/vacancy diffusion at the dislocation cores. We note that the intensity values measured in our HRTEM images may not be linearly related to the number of the atoms within an individual atomic column due to unavoidable misalignment of the sample or residual optical lens aberrations.

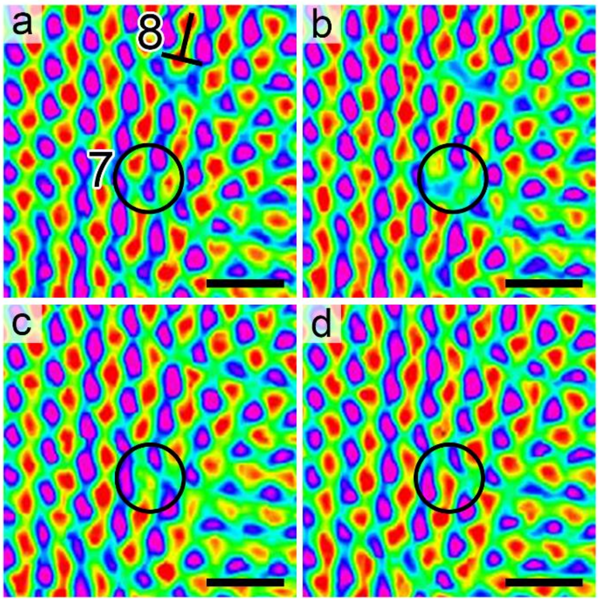

Supplementary Figure 12. Experimental HRTEM images with intensity shown in false color. As circled in black, the red spots corresponding to atomic columns at the dislocation core in (a) became faint in (b) due to the massive atom diffusion away. Accordingly, affected by atom diffusion and rearrangement, the color of the blue spots corresponding to the channels became lighter simultaneously. In (c) and (d), two red spots merged into a single one, indicating the completion of dislocation climb. Scale bar: 0.5 nm.

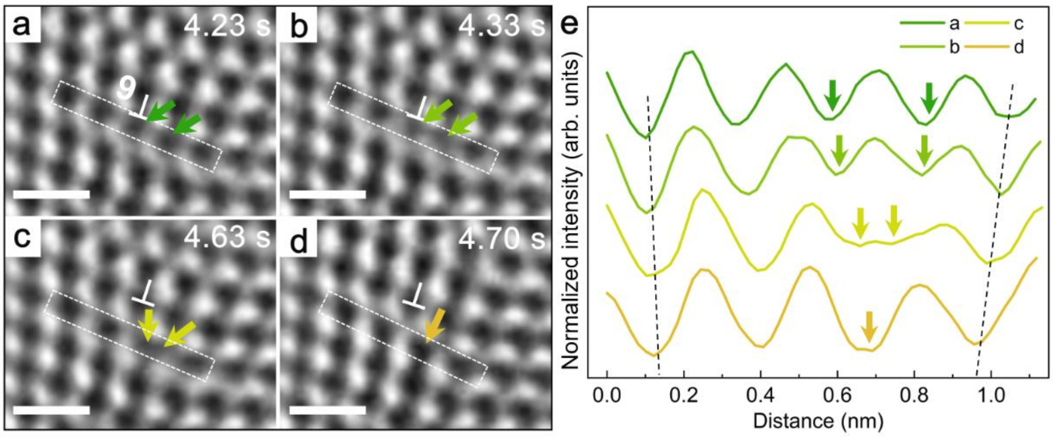

Supplementary Figure 13. Additional example of two atomic columns involved in the reconstruction of the core of a positively-climbing GB dislocation. The contrast intensity profiles in (e) are extracted along the dotted rectangles in (a)-(d). Scale bar: 0.5 nm.

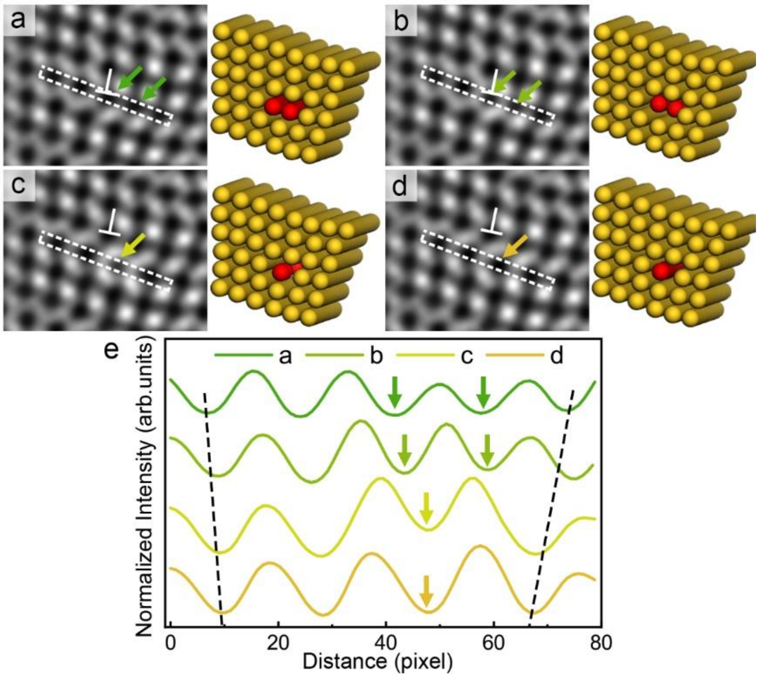

Supplementary Figure 14. Simulated HRTEM images based on the reconstructed atomic configurations at the core of a climbing dislocation and the corresponding profile evolution. (a)-(d) Simulated HRTEM images (left) and corresponding atomic configurations (right) during climb of a GB dislocation. In the simulation, the defocus value is set as 10 nm and the sample has 16 layers of $(1\bar{1}0)$ plane along the $[1\bar{1}0]$ direction. Dark spots in the simulated HRTEM images represent atomic columns. Red atomic columns are used to represent the dislocation core in the reconstructed atomic configuration (right) in (a). A half amount of atoms are being removed from the two red columns in the dislocation core in (b), followed by the merging process in (c). Two red columns merge into one column in (d), indicating the completion of dislocation climb by one atomic layer. (e) The normalized contrast intensities along the dotted rectangles in (a)-(d), showing the same dislocation climb process as the experimental results.

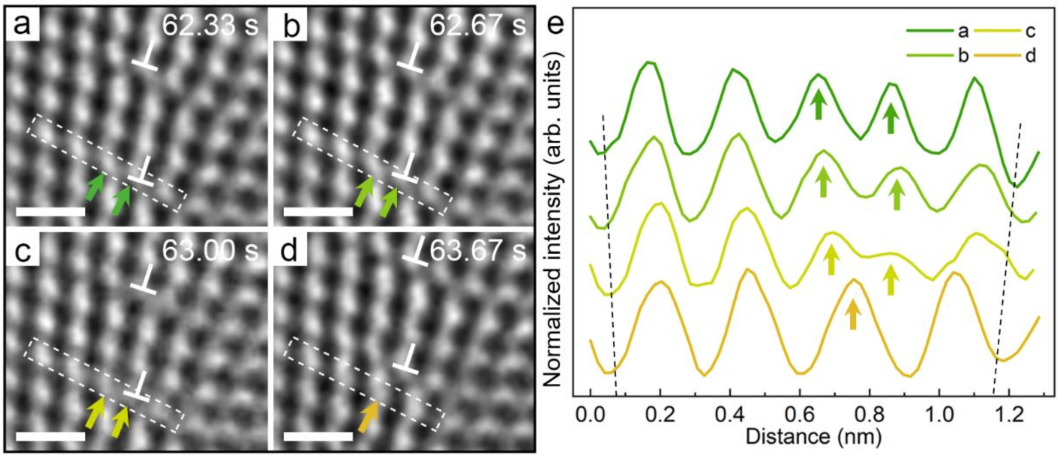

Supplementary Figure 15. An alternative analysis of dislocation climb by taking the bright spots in HRTEM images as atomic columns. (a)-(d) *In situ* HRTEM images showing dislocation climb. (e) Contrast intensity profiles extracted along the dotted rectangles in (a)-(d). Peaks pointed by arrows in the line profile *a* to *c* indicate the gradually decrease of contrast intensity. The decreased distance between the two peaks indicates the merging process of the two atomic columns at the dislocation core. The appearance of a single peak (atomic column) with the concomitant of disappearance of the two previous peaks (atomic columns) in the line profile *d* indicates the completion of dislocation climb by one atomic layer. Scale bar: 0.5nm.

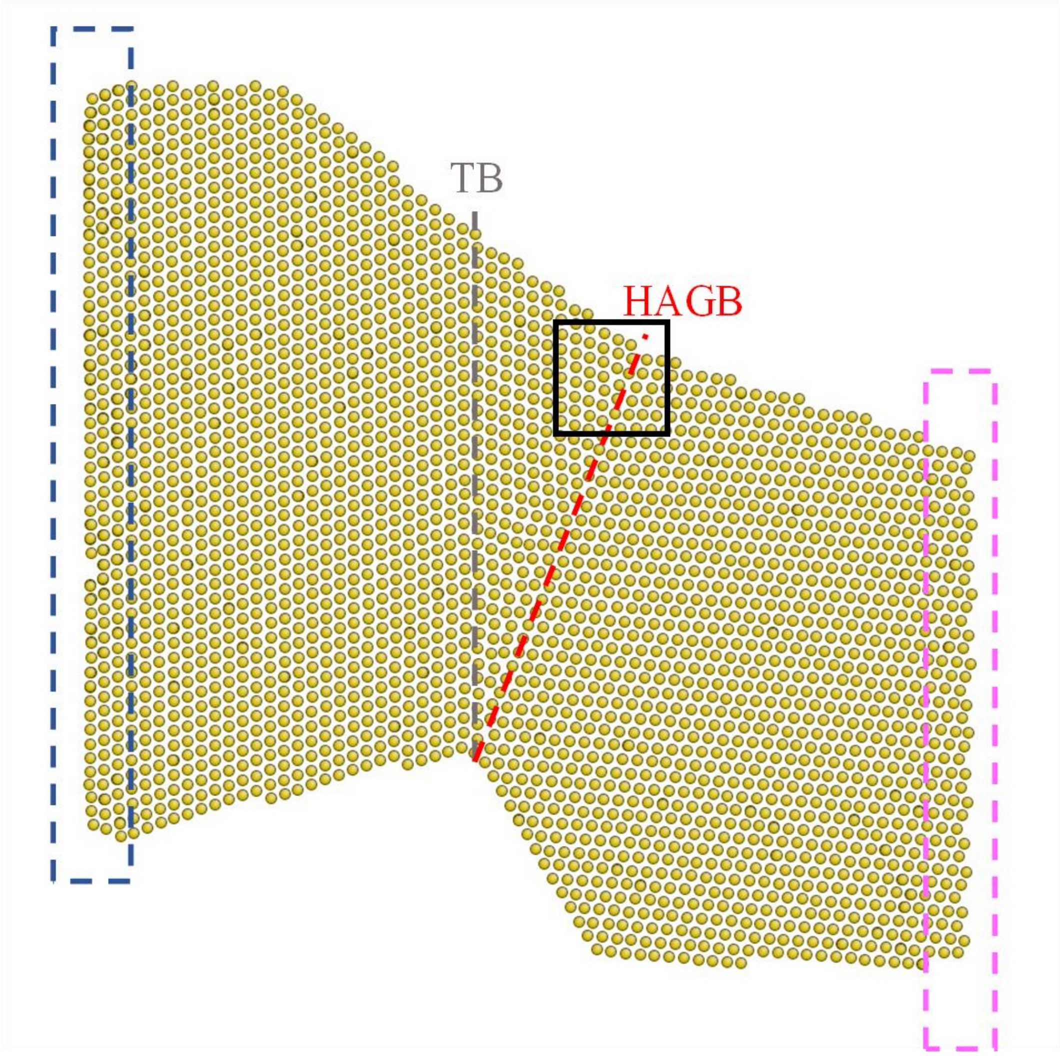

Supplementary Figure 16. GCMC simulation setup showing the atomic configuration of the Au ligament reconstructed from the HRTEM image in Fig. 1a. TB and HAGB are marked by grey and red dashed lines, respectively. During GCMC simulation, atoms on the left side (blue box) of the reconstructed Au ligament were fixed and atoms on the right side (pink box) were subjected to an applied bending load.

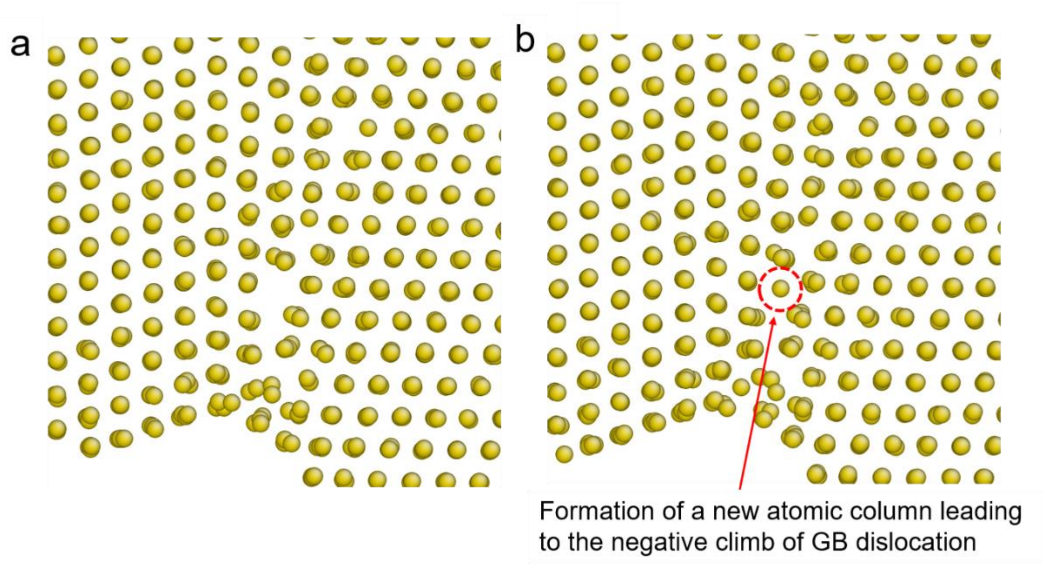

Supplementary Figure 17. GCMC simulation result of negative climb of a GB dislocation. (a) The atomic structure before climb. (b) The atomic structure after climb. As marked by the red dashed circle, a new atomic column formed, leading to the negative climb of the GB dislocation.

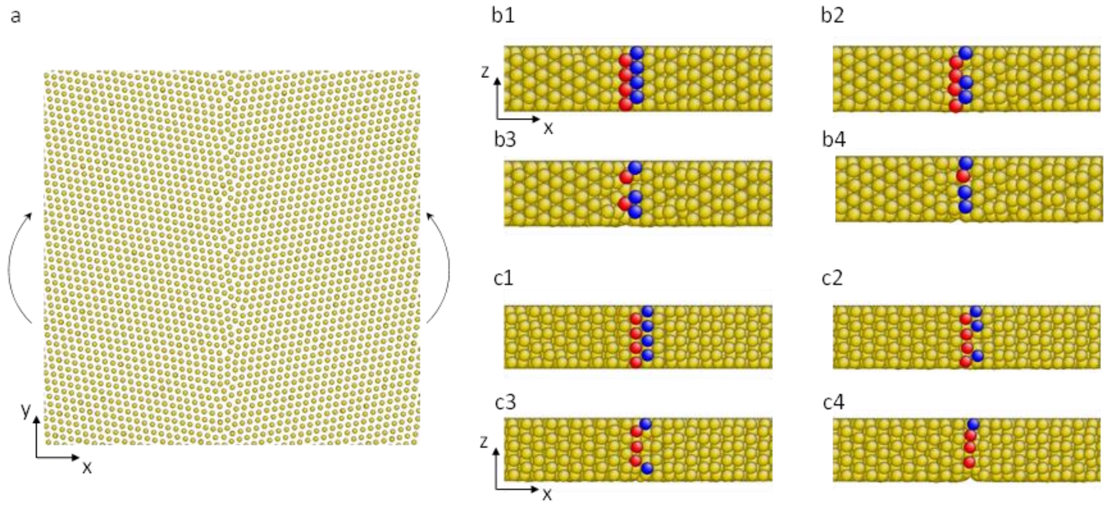

Supplementary Figure 18. GCMC simulation of dislocation climb at a symmetric GB under a symmetric bending load. (a) The applied bending load is indicated by the arrows. The top view shows the x-y section of the bicrystal. (b1)-(b4) One example of GCMC snapshots showing the merging of two atomic columns into one at the core of a climbing GB dislocation. The side view shows the x-z section of the bicrystal. (c1)-(c4) Another example of GCMC snapshots showing the merging of two atomic columns into one at the core of a climbing GB dislocation. The two merging atomic columns are colored by red and blue, respectively, for guiding eyes. The side view shows the x-z section of the bicrystal.

Supplementary Note 5. The maximum increase of temperature under electron irradiation was estimated by Fisher's model[8]:

$$\Delta T = \frac{I}{\pi\kappa e}\left(\frac{\Delta E}{d}\right)\ln\frac{b_s}{r_0} \quad (14)$$

where I is the total beam current, κ is the thermal conductivity, e is the electron charge, b_s is the sample radius, r_0 is the effective beam radius, and ΔE is the total energy loss per electron in a sample with a thickness of d. The total beam current $I = J\pi r_0^2$, where J is the current intensity. Because energy loss in the sample was small compared to the initial energy (200 keV), the term $\Delta E/d$ was equal to the stopping power of electrons, dE/dx, which was calculated from the Bethe-Bloch equation[9]:

$$-\frac{dE}{dx} = \frac{2\pi Z\rho\left(\frac{e^2}{4\pi\varepsilon_0}\right)^2}{m\upsilon^2}\{\ln\left[\frac{E(E+mc^2)^2\beta^2}{2I_e^2mc^2}\right] + (1-\beta^2) - \left(1-\sqrt{1-\beta^2}+\beta^2\right)\ln2 + \frac{1}{8}\left(1-\sqrt{1-\beta^2}\right)^2\} \quad (15)$$

where Z is the atomic number of the target element, ρ is the atomic density, ε_0 is the dielectric constant, m is the electron rest mass, υ is the electron velocity, E is the electron energy, c is the speed of light, I_e is the average excitation energy of electrons in the

target, and $\beta = \upsilon/c$. In the present study, the acceleration voltage is 200 kV, such that $\beta = 0.6946$ and $\upsilon = 2.0837\times10^{8}$ m s^{-1}.

For Au, Z = 79, $\rho = 4.79\times10^{29}$ m^{-3} (the mass density is 19320 kg m^{-3}), $I_e = 10Z = 790$, κ = 301 W m^{-1} K^{-1}, $m = 9.1\times10^{-31}$ kg, e = 1.6×10^{-19} C, $c = 3\times10^{8}$ m s^{-1}, $\varepsilon_0 = 8.85\times10^{-12}$ F m^{-1}. By using these two equations, the maximum temperature increase was estimated to be 3.8 K in the Au region in our experiments with I = 2.5 nA, b_s = 1.5 mm and r_0 = 100 nm. Hence, the effect of electron beam irradiation only leads to a few degrees above room temperature, which has negligible contributions to the dislocation climb behavior.

Supplementary Note 6. Under electron-beam irradiation, the maximum energy transferred to a Au atom in a perfect lattice can be estimated as[10]

$$\Delta E_{\max} = \frac{2E\,(E + 2m_e c^2)}{Mc^2} \tag{16}$$

Here E is the electron energy, m_e is the electron mass, c is the speed of light and M is the mass of the Au atom. The maximum energy that an electron beam can transfer to the Au atom is ~ 2.66 eV when the TEM is operated at 200 kV, which is much lower than the threshold displacement energy (i.e., a minimum amount of kinetic energy transferred to a lattice atom that results in the formation of a point defect) of ~ 36 eV for Au[11]. Hence, when the TEM is operated at 200 kV, the knock-on displacement of electron beam on Au atoms is negligible.

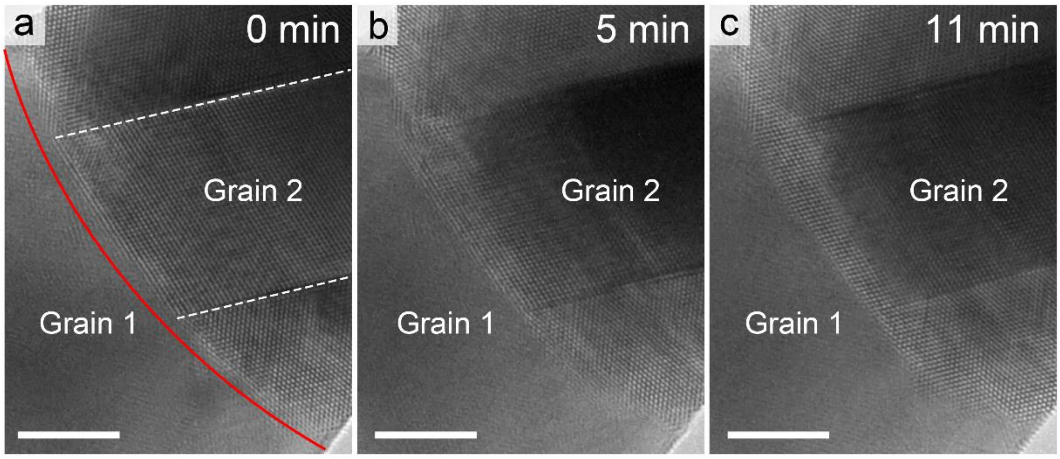

Supplementary Figure 19. *In situ* HRTEM images of a GB in an Au ligament under electron beam irradiation without applied straining. (a) HRTEM image of the Au ligament at 0 min. The GB is marked by the red line and the TBs in Grain 2 are marked by the white dashed lines. (b)-(c) HRTEM images of the Au ligament under electron beam irradiation for 5 min and 11 min, respectively. It is clear that no significant events of dislocation climb/glide, GB evolution or grain rotation occur. The GBs and dislocations in Au ligaments under 200 keV electron beam irradiation are stable. Scale bar: 5 nm.

Supplementary Table 1. Parameters used in the HRTEM image simulations.

Acceleration voltage	200 kV
Spherical aberration coefficient	-1.15 μm
Defocus spread	3 nm
Beam convergence	2 mrad
Defocus value (underfocus)	7.5 nm; 10 nm; 12.5 nm

Supplementary Table 2. Maximum climb velocity of dislocations "1" - "9".

Dislocation number	1	2	3	4	5	6	7	8	9
Maximum velocity (nm s^{-1})	-1.40×10^{-3}	0	1.40×10^{-3}	17.2	15.6	7.09×10^{-1}	1.28×10^{-1}	2.48	3.23×10^{-1}

Supplementary References

1. Ishizuka, K. Contrast transfer of crystal images in TEM. *Ultramicroscopy* **5**, 55-65 (1980).
2. Ishizuka, K. Multislice formula for inclined illumination. *Acta Cryst. A* **38**, 773-779 (1982).
3. Hirth, J. & Lothe, J. *Theory of Dislocations.* 2nd, (Wiley, New York, 1982).
4. Frank, F. C. *A symposium on the plastic deformation of crystalline solids* (Office of Naval Research, Pittsburgh 150, 1950).
5. Jin, Z. H.*, et al.* Interactions between non-screw lattice dislocations and coherent twin boundaries in face-centered cubic metals. *Acta Mater.* **56**, 1126-1135 (2008).
6. Wang, Y. B. & Sui, M. L. Atomic-scale in situ observation of lattice dislocations passing through twin boundaries. *Appl. Phys. Lett.* **94**, 021909 (2009).
7. Lynch, D., Moodie, A. F. & O'keefe, M. n-Beam lattice images. V. The use of the charge-density approximation in the interpretation of lattice images. *Acta Cryst. A* **31**, 300-307 (1975).
8. Fisher, S. B. On the temperature rise in electron irradiated foils. *Radiat. EIT.* **5**, 239-243 (1970).
9. Jenčič, I., Bench, M. W., Robertson, I. M. & Kirk, M. A. Electron-beam-induced crystallization of isolated amorphous regions in Si, Ge, GaP, and GaAs. *J. Appl. Phys.* **78**, 974-982 (1995).
10. Banhart, F. Irradiation effects in carbon nanostructures. *Rep. Prog. Phys.* **62**, 1181-1221 (1999).
11. Vajda, P. Anisotropy of electron radiation damage in metal crystals. *Rev. Mod. Phys.* **49**, 481-521 (1977).
