# Supplementary material for: In situ atomic-scale observation of dislocation climb and grain boundary evolution in nanostructured metal
Source: Nat Commun. 2022 Jul 18;13:4151. doi: 10.1038/s41467-022-31800-8 (PMC9293973; doi:10.1038/s41467-022-31800-8)
Supplement: Supplementary file 3 — Description of Additional Supplementary Files [file 41467_2022_31800_MOESM3_ESM.pdf]

Description of Additional Supplementary Files

File Name: Supplementary Movie 1
Description: In situ bending deformation of an Au ligament, recorded at a rate of 30 frames per second and played at 5x speed.

File Name: Supplementary Movie 2
Description: Fast dislocation climb at the final stage of deformation, recorded at a rate of 30 frames per second and played at 0.2x speed.

File Name: Supplementary Movie 3
Description: Positive dislocation climb with the evolution of dislocation core, recorded at a rate of 30 frames per second and played at 0.125x speed.

File Name: Supplementary Movie 4
Description: Negative dislocation climb with the evolution of dislocation core, recorded at a rate of 30 frames per second and played at 0.2x speed.

File Name: Supplementary Movie 5
Description: Another example of positive dislocation climb and the evolution of dislocation core, recorded at a rate of 30 frames per second and played at 0.125x speed.
